# The identification and cognitive correlation of perfusion patterns measured with arterial spin labeling MRI in Alzheimer’s disease

**DOI:** 10.1186/s13195-023-01222-9

**Published:** 2023-04-10

**Authors:** Meng Meng, Fang Liu, Yilong Ma, Wen Qin, Lining Guo, Shichun Peng, Marc L. Gordon, Yue Wang, Nan Zhang

**Affiliations:** 1 Department of Neurology, Tianjin Medical University General Hospital Airport Site, Tianjin, China; 2 Department of Neurology, Tianjin Neurological Institute, Tianjin Medical University General Hospital, 154, Anshan Road, Tianjin, 300052 China; 3 Center for Neurosciences, Institute of Molecular Medicine, The Feinstein Institutes for Medical Research, Northwell Health, Manhasset, NY USA; 4 Department of Molecular Medicine, Donald and Barbara Zucker School of Medicine at Hofstra-Northwell, Hofstra University, Hempstead, NY USA; 5 Department of Radiology and Tianjin Key Laboratory of Functional Imaging, Tianjin Medical University General Hospital, Tianjin, China; 6 The Litwin-Zucker Research Center, The Feinstein Institutes for Medical Research, Northwell Health, Manhasset, NY USA; 7 Departments of Neurology and Psychiatry, Donald and Barbara Zucker School of Medicine at Hofstra-Northwell, Hofstra University, Hempstead, NY USA

**Keywords:** Arterial spin labeling, Cerebral blood flow, Alzheimer’s disease, Principal component analysis, Scaled subprofile model

## Abstract

**Background:**

Vascular dysfunction, including cerebral hypoperfusion, plays an important role in the pathogenesis and progression of Alzheimer’s disease (AD), independent of amyloid and tau pathology. We established an AD-related perfusion pattern (ADRP) measured with arterial spin labeling (ASL) MRI using multivariate spatial covariance analysis.

**Methods:**

We obtained multimodal MRI including pseudo-continuous ASL and neurocognitive testing in a total of 55 patients with a diagnosis of mild to moderate AD supported by amyloid PET and 46 normal controls (NCs). An ADRP was established from an identification cohort of 32 patients with AD and 32 NCs using a multivariate analysis method based on scaled subprofile model/principal component analysis, and pattern expression in individual subjects was quantified for both the identification cohort and a validation cohort (23 patients with AD and 14 NCs). Subject expression score of the ADRP was then used to assess diagnostic accuracy and cognitive correlations in AD patients and compared with global and regional cerebral blood flow (CBF) in specific areas identified from voxel-based univariate analysis.

**Results:**

The ADRP featured negative loading in the bilateral middle and posterior cingulate and precuneus, inferior parietal lobule, and frontal areas, and positive loading in the right cerebellum and bilateral basal areas. Subject expression score of the ADRP was significantly elevated in AD patients compared with NCs (*P* < 0.001) and showed good diagnostic accuracy for AD with area under receiver–operator curve of 0.87 [95% CI (0.78–0.96)] in the identification cohort and 0.85 in the validation cohort. Moreover, there were negative correlations between subject expression score and global cognitive function and performance in various cognitive domains in patients with AD. The characteristics of the ADRP topography and subject expression scores were supported by analogous findings obtained with regional CBF.

**Conclusions:**

We have reported a characteristic perfusion pattern associated with AD using ASL MRI. Subject expression score of this spatial covariance pattern is a promising MRI biomarker for the identification and monitoring of AD.

**Supplementary Information:**

The online version contains supplementary material available at 10.1186/s13195-023-01222-9.

## Introduction

Although biomarker development, in particular focusing on amyloid-β (Aβ) and paired helical filament tau, is improving the diagnostic efficacy of Alzheimer’s disease (AD), the invasive procedure of cerebrospinal fluid (CSF) testing and the limited availability of positron emission tomography (PET) impede their wide clinical application. Moreover, the current biomarker system for AD diagnosis is not absolutely specific or comprehensive. Patients with other conditions, such as dementia with Lewy bodies (DLB) and cerebral amyloid angiopathy, or even cognitively healthy individuals could also exhibit positive results for Aβ and tau on PET scans [1]. On the other hand, the pathophysiological process of AD is quite complex and potentially heterogeneous and may not be limited to Aβ and tau. The National Institute on Aging and the Alzheimer’s Association (NIA-AA) Research Framework indicated that other biomarkers may be incorporated in the AT(N) system in the future, such as TDP-43, α-synuclein, biomarkers of neuroinflammation, and vascular biomarkers in particular [2].

There is abundant evidence that brain vasculature plays an important role in the pathogenesis and progression of AD, even independent of Aβ and tau and vascular risk factors [3]. Moreover, an updated hypothetical model of AD biomarkers has suggested that vascular dysfunction, such as changes in the cerebral blood flow (CBF) and blood–brain barrier, may contribute to the initial stage of the pathophysiological process in AD, even before Aβ and tau pathology [4]. As an indicator of not only neurovascular uncoupling but also brain dysfunction, CBF assessment is a promising tool for early diagnosis and disease monitoring in patients with AD [5].

Arterial spin labeling (ASL) is a non-invasive MRI technique to quantitatively measure CBF using arterial blood water as an endogenous tracer, which is less time-consuming, and more feasible and repeatable compared with traditional perfusion measurements, such as PET and single photon emission computed tomography (SPECT) [6]. Alsop et al. first reported significant global and regional CBF reduction measured with continuous ASL in patients with AD [7]. While not entirely consistent, subsequent ASL studies have observed robustly decreased CBF in the cingulate, precuneus, parietal lobes, and inferior frontal regions [6].

Although previous studies suggested that ASL is a reliable measure of neurovascular dysfunction in AD, they employed voxel-by-voxel univariate analytical methods that ignored intrinsic functional correlations between anatomical structures of interest. Scaled subprofile model (SSM) is a multivariate analysis method based on principal component analysis (PCA) to identify significant spatial covariance patterns in functional brain images [8]. Indeed, this method has been used to establish disease-related metabolic patterns of AD [9–11] and other neurodegenerative diseases on the basis of ^18^F-fluorodeoxyglucose (FDG) PET.

A preliminary study including a small sample with clinically diagnosed AD identified an AD-related perfusion pattern (ADRP) using continuous ASL, which featured negative loading mainly in temporal and parietal brain areas [12]. However, there have been few subsequent investigations in larger and independent cohorts, in particular in patients with biomarker confirmed AD. We previously identified an age-related perfusion pattern from pseudo-continuous ASL (pCASL) data using the method of SSM/PCA and demonstrated its individual evaluation according to subject expression score [13]. In this study, we established an ADRP with SSM/PCA in patients with AD whose diagnosis was supported by amyloid PET scan. We then tested and cross-validated the expression of this ADRP as an MRI perfusion biomarker for AD diagnosis and objective assessment of clinical severity with cognitive function in different domains. Finally, we compared the characteristics of the ADRP and its subject expression scores with regionally specific CBF values and cognitive correlations using complementary univariate analysis, which was performed independently from the SSM/PCA procedure.

## Methods

### Participants

Thirty-four patients with AD and 34 age- and sex-matched normal controls (NC) were first recruited as the identification cohort at baseline from our longitudinal MRI study of Alzheimer’s disease and subcortical ischemic vascular dementia (ChiCTR1900027943), which was approved by the Ethics Committee of Tianjin Medical University General Hospital, China. Written informed consent was obtained from all participants and their legally designated representatives prior to inclusion. All participants underwent a standard clinical evaluation, including demographics and medical history, physical and neurologic examinations, comprehensive neuropsychological assessments, laboratory tests, carotid duplex ultrasound and transcranial doppler (TCD), and brain MRI. Two patients with AD were excluded from the final analysis because of image artifacts caused by severe head motion, and 2 NCs were excluded because intracranial tumor was found on MRI scan. This cohort of 32 AD and 32 NC subjects were used for identification of the ADRP. For validation of the ADRP expression, another independent cohort including 23 patients with AD and 14 NCs was also recruited at baseline from the same longitudinal study. The identification cohort consisted of subjects who were recruited before Dec. 2019 while the validation cohort included additional subjects enrolled subsequently between Jan. 2020 and Apr. 2022. No subject was found to have moderate or severe stenosis in large vessels by carotid duplex ultrasound, TCD, or cranial MR angiography.

AD patients met the diagnostic criteria for major neurocognitive disorder according to the Diagnostic and Statistical Manual of Mental Disorders, Fifth Edition [14] and the research diagnostic criteria for typical AD according to the International Working Group-2 [15]. Inclusion criteria for AD patients were (1) age 50–85 years, ≥ 3 years of education; (2) presence of an early and prominent episodic memory impairment; (3) Clinical Dementia Rating (CDR) [16] score = 1–2, Mini-Mental State Examination (MMSE) [17] score = 10–26; (4) no evidence of clinically significant cerebrovascular lesions or extensive white matter hyperintensities (WMH) on MRI, Fazekas score [18] < 2; and (5) a positive result for Aβ deposition measured with ^11^C-Pittsburgh Compound B (^11^C-PiB) PET according to our previously described protocol [19]. Patients whose cognitive impairment was potentially caused by other neurological diseases, mental disorders or medical conditions, such as frontotemporal lobar degeneration, Parkinson’s disease dementia or DLB, vascular dementia, multiple sclerosis, hydrocephalus, thyroid dysfunction, vitamin B_12_ deficiency, HIV infection, neurosyphilis, alcohol or drug abuse, or severe depression, were excluded. Although the 17 item-Hamilton Depression Scale (HAM-D) [20] was not used as an initial exclusion criterion, none of the AD patients in this study had a HAM-D score greater than 10.

Inclusion criteria for NCs included (1) age 50–85 years, ≥ 3 years of education; (2) no subjective cognitive decline complaints and normal performance in each cognitive domain of objective neuropsychological tests; (3) CDR score = 0, MMSE score > 26, HAM-D score < 17; and (4) no clinically significant brain atrophy (medial temporal lobe atrophy score [21] = 0–1 for subjects < 75 years old or 0–2 for subjects ≥ 75 years old) or cerebrovascular lesions (Fazekas score < 2) on brain MRI.

### Neuropsychological assessment

A neuropsychological battery was performed to evaluate various cognitive domains in 52 of 55 patients with AD and all NCs as previously described [22, 23], including the Chinese version of the Rey Auditory Verbal Learning Test (AVLT), the Brief Visuospatial Memory Test-Revised (BVMT-R), the Symbol Digit Modalities Test (SDMT), the Trail Making Test-A (TMT-A) and TMT-B, the Stroop color–word test, the Animal Verbal Fluency Test (AFT), the Controlled Oral Word Association Test (COWAT), the Boston Naming Test (BNT), and the Benton Judgment of Line Orientation (JLO). Raw scores were converted to z-scores using the mean and standard deviation of all NCs included in this study. Five main cognitive domains were calculated: (1) memory composite = average z-score of total learning, delayed recall and recognition on the AVLT and the BVMT-R; (2) attention and information processing speed composite = average of the SDMT and the TMT-A; (3) executive function composite = average of the TMT-B and the Stroop color–word test; (4) language composite = average of the AFT, the COWAT and the BNT; and (5) visuospatial function = z-score of the JLO. Three patients with AD only received the MMSE but refused further cognitive assessment.

### MRI acquisition

All subjects did not take any medication that might influence CBF regulation within the previous 2 weeks and refrained from alcohol, caffeine, and nicotine for at least 6 h prior to MRI measurement. The imaging acquisition was performed on a 3.0-Tesla MRI scanner (Discovery MR750, General Electric, Milwaukee, WI, USA), using a 64-channel phased array head coil. The coronal T1-weighted 3D brain volume sequence was first performed to serve as a template for co-registration with ASL imaging data: echo time/repetition time (TE/TR): 3.2 ms/8.2 ms, flip angle (FA): 12°, field of view (FOV): 256 × 256 × 188mm^3^, matrix size: 256 × 256, NEX = 1, slice thickness: 1.0 mm, number of slices: 188. The 3D pseudo-continuous ASL series was prepared to measure whole brain perfusion using a 3D fast spin-echo acquisition and background suppression: TE/TR: 11.1 ms/5046 ms, labeling duration: 1450 ms, post labeling delay (PLD): 2025 ms, FA: 111°, matrix size: 128 × 128, FOV: 240 × 240 × 150 mm^3^, arms = 8, acquisition points = 512, slice thickness: 3 mm, number of slices: 50, total scan time = 4 min and 53 s. The labeling plane was placed 20 mm inferior to the lower edge of the imaging volume. A proton density image was also acquired at the same time to quantify CBF from the ASL series. During the resting state scan of ASL, participants had their ears plugged, and were instructed to keep their eyes closed, not to think of anything in particular, not to fall asleep, and to remain still during each series. Foam padding was inserted around the sides of the head and the forehead to minimize patient motion.

### Imaging data preprocessing

All images were preprocessed with statistical parametric mapping (SPM12, Institute of Neurology, London, UK) software running in MATLAB (Version R2015a; MathWorks, Natick, MA, USA) on a Windows computer. All images underwent manual quality control checks by a trained investigator for image quality and successful co-registration. The ASL MRI images were automatically converted into CBF maps using Functool software (version 9.4, GE Medical Systems) on an Advantage Windows workstation to identify changes in global and regional CBF. Image preprocessing was conducted as follows: (1) CBF images were registered to structural MRI images (linear deformation, 4th Degree B-Spline); (2) the structural images were normalized to the standard Montreal Neurological Institute (MNI) brain template and segmented into probability maps of grey matter (GM), white matter (WM), and CSF; (3) the CBF images were normalized using the parameters determined from the structural images and multiplied by a binary brain tissue mask only consisting of GM and WM; (4) normalized CBF maps were then smoothed by using a Gaussian kernel with a full width at half maximum (FWHM) of 10 mm to minimize registration errors, ensure isotropic smoothness, and satisfy the Random Field Theory assumptions used for the voxel-based analyses. All processed images of CBF and tissue maps have a matrix dimension of 121 × 145 × 121 and a voxel size of 1.5 × 1.5 × 1.5 mm^3^ in the MNI space.

### Identification and validation of ADRP using SSM/PCA

Multivariate spatial covariance technique was applied to assess subject-by-voxel effects on CBF maps in all participants from the identification cohort with SSM/PCA toolbox freely (available http://www.feinsteinneuroscience.org) [24]. This was conducted using CBF maps obtained with ASL MRI without applying log-transformation and within a GM mask. This mask was created with a threshold of ≥ 0.3 from the probabilistic tissue map of GM (voxel size = 1 × 1 × 1 mm^3^) available under the TPM folder in SPM and resampled into the CBF maps which had already been brought into the MNI space as noted above. Once a significant pattern has been identified, its expression in a given subject can be determined from that individual’s scan. The corresponding subject expression scores reflect the degree to which each subject expresses these patterns individually. Multiple regression analysis was used to identify the best set of SSM component patterns singly or in combination that can maximally discriminate AD from NCs. We saved the first 6 patterns that accounted for > 60% of subject × voxel variance in the SSM/PCA operation and identified a subset of these 6 patterns by binary logistic regression analysis with group as the dependent variable and subject expression scores for the patterns as independent variables. These component patterns and their subject expression scores were linearly combined using the regression parameters to define an ADRP and corresponding subject expression scores. Subject expression scores were then z-transformed using mean and standard deviation of the NC group. The reliability of the ADRP was evaluated by a bootstrapping resample scheme described previously [11]. Coordinates of the resulting topography were reported in the standard MNI anatomical space. Significant regions were localized by Talairach–Daemon software (Research Imaging Center, University of Texas Health Science Center, San Antonio, TX, USA). Nearest GM locations were reported for all these regions. The PCA maps were overlaid on a standard T1-weighted MRI brain template in stereotaxic space.

Subject expression score was also obtained for each CBF image in the validation cohort on a prospective single-case basis using a voxel-based algorithm on SSM/PCA toolbox. The resulting score was z-transformed with respect to the NC group in the identification cohort.

### Brain mapping analysis with SPM

Univariate analysis was performed in the identification cohort using SPM12 software. This was done independently from the aforementioned multivariate analysis, but within the same GM mask described above. CBF differences between the AD and NC groups were compared with a two-sample *t*-test model, using age and sex as covariates. With a voxel-level peak threshold of *P* < 0.05 (family wise error-corrected, FWE-corrected) over whole brain regions, we primarily identified clusters > 317 voxels (voxel size = 1.5 mm × 1.5 mm × 1.5 mm) for the analysis of absolute CBF and clusters > 66 voxels after adjusting for global values with ANCOVA. Coordinates reporting, anatomical localization, and display of the CBF differences were performed using the same procedures as for the PCA maps described above.

To quantify CBF changes in specific cortical regions, we used a 4-mm radius spherical volume of interest (VOI) centered at the peak voxel of clusters that were significant in the SPM analyses. We then obtained the relative CBF values by calculating the ratio of the VOI values to the global CBF values in all participants from the identification cohort with SPM12.

### Statistical analysis

Statistical analysis on vector data was performed using SPSS20.0 (SPSS, Inc., Chicago, IL, USA) unless otherwise specified. All the tests were two-tailed, and values of *P* < 0.05 were regarded as significant. Demographics of patients with AD and NCs were analyzed using Pearson chi-square test for categorical variables or independent-sample *t*-test for continuous variables.

The differences in subject expression scores of the ADRP, and global CBF value and relative regional CBF values between the AD and NC groups, were compared with two-sample *t*-tests. Then receiver operator characteristic (ROC) analysis was generated to assess the performance of the modality as a classifier predicting the disease status (AD or NC), using GraphPad Prizm version 8.0 for Windows (GraphPad Software, San Diego, CA, USA). The area under the curve (AUC) and the optimal cutoff point were determined to indicate the performance of the ADRP and CBF value in the diagnosis of AD.

To clarify whether the ADRP subject expression and CBF value correlated with cognitive performance and disease severity, the relationships between subject expression scores, global CBF value and relative regional CBF values, and z-scores of the MMSE and cognitive composite were further analyzed with linear regression in patients with AD.

## Results

### Demographics and clinical profile of the study population

Demographic and clinical characteristics of all participants are summarized in Table 1. There was no significant difference in age or sex between the AD group and the NC group in either the identification cohort or the validation cohort. All participants were right-handed. AD patients had fewer years of education (10.19 ± 4.14 vs 12.75 ± 3.49, *t* = 2.677, *P* = 0.009) than NCs in the identification cohort.

**Table 1:** Demographics and cognitive scores of patients with AD and NCs

	Identification cohort			Validation cohort		
	AD group	NC group	*P*	AD group	NC group	*P*
	*N* = 32	*N* = 32		*N* = 23	*N* = 14	
Age^a^, years	66.00 (7.58)	64.97 (5.52)	0.536	69.00 (6.92)	69.79 (6.09)	0.645
Sex, F/M	23/9	22/10	0.784	15/8	7/7	0.493
Education, years	10.19 (4.14)	12.75 (3.49)	0.009	10.70 (2.44)	12.00 (2.80)	0.145
Handedness, R/L/A	32/0/0	32/0/0	—	23/0/0	14/0/0	—
MMSE score^a^	16.75 (4.54)	28.19 (1.23)	< 0.001	20.35 (3.94)	27.57 (1.09)	< 0.001
Memory	− 3.44 (0.69)	0.08 (0.71)	< 0.001	− 3.41 (0.90)	− 0.18 (0.91)	< 0.001
Processing speed	− 2.61 (1.42)	0.19 (0.90)	< 0.001	− 2.04 (1.42)	− 0.43(0.80)	< 0.001
Executive function	− 2.00 (1.26)	0.17 (0.62)	< 0.001	− 1.74 (0.77)	− 0.39 (0.91)	< 0.001
Language^a^	− 1.06 (0.49)	0.01 (0.43)	< 0.001	− 0.76 (0.54)	− 0.03 (0.34)	< 0.001
Visuospatial function^a^	− 3.17 (2.31)	− 0.04 (1.06)	< 0.001	− 1.37 (1.78)	0.10 (0.88)	0.007

Data are provided as mean (SD) unless otherwise specified. z-scores for different cognitive domains were calculated from raw scores of neuropsychological assessments in reference to the mean and standard deviation of all NCs. Memory = average of total learning, delayed recall and recognition on the AVLT and the BVMT-R; processing speed = average of the SDMT and the TMT-A; executive function = average of the TMT-B and the Stroop color–word test; language = average of the AFT, the COWAT and the BNT; visuospatial function = the JLO. Data for cognitive domains was missing from 3 AD patients

*AD* Alzheimer’s disease, *NC* normal control, *MMSE* Mini-Mental State Examination, *R* right-handed, *L* left-handed, *A* ambidextrous

^a^There was a significant difference between the identification cohort and the validation cohort. NC group in the validation cohort was older than that in the identification cohort (*P* = 0.011). AD group in the validation cohort had higher MMSE score (*P* = 0.004) and z-scores of language (*P* = 0.043) and visuospatial function (*P* = 0.003) domains than those in the identification group

MMSE score (identification cohort: 16.75 ± 4.54 vs 28.19 ± 1.23, *t* = 13.75, *P* < 0.001; validation cohort: 20.35 ± 3.94 vs 27.57 ± 1.09, *t* = 6.676, *P* < 0.001) and z-scores of all cognitive domains were lower in AD patients than NCs in both cohorts. However, AD patients in the validation cohort had higher MMSE score (*t* = 3.059, *P* = 0.004) and z-scores of language (*t* = 2.081, *P* = 0.043) and visuospatial function (*t* = 3.099, *P* = 0.003) domains than those in the identification cohort.

### CBF pattern identified by multivariate analysis

**Fig. 1:**
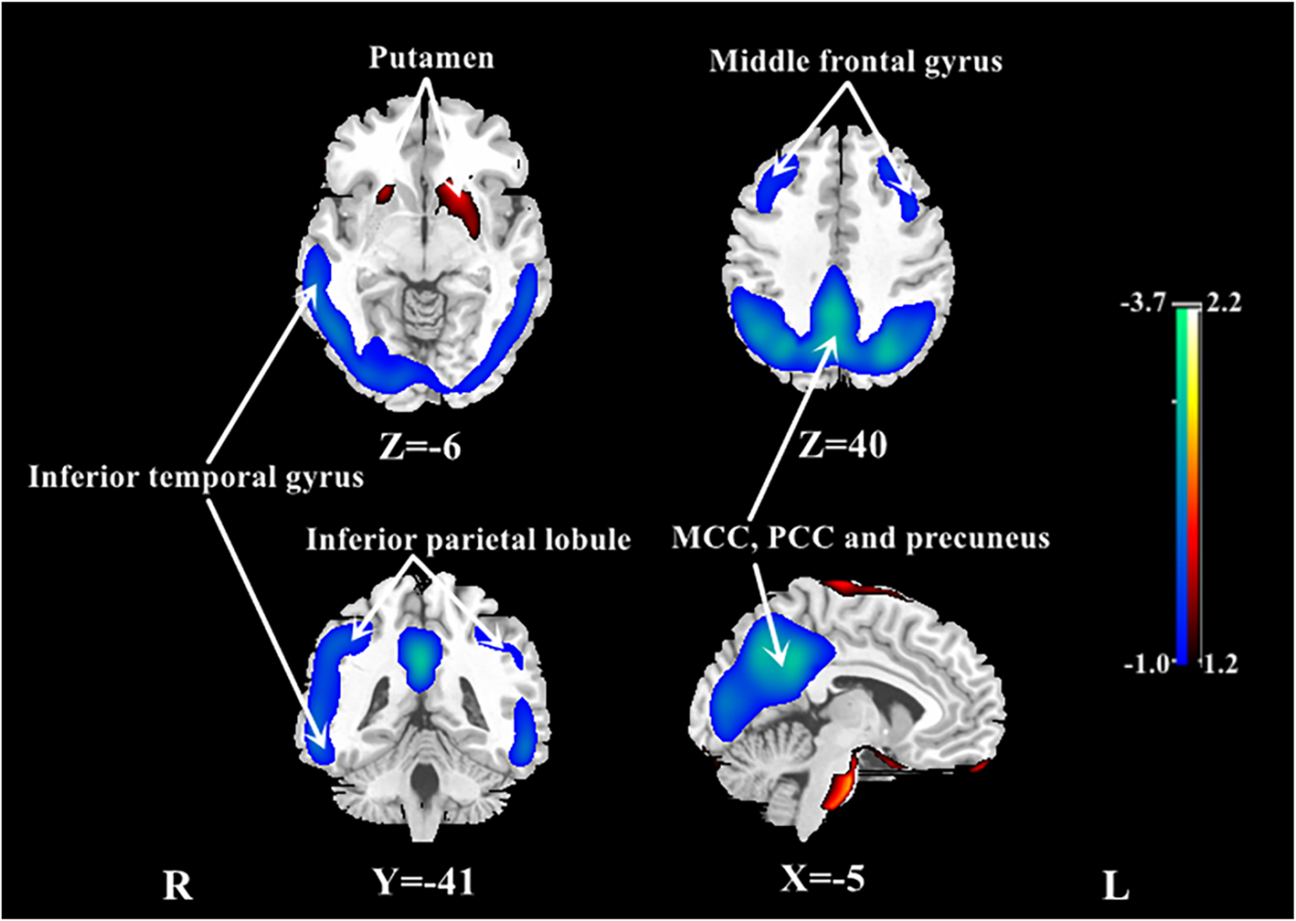
Regional topographies of ADRP measured with ASL MRI. The ADRP was identified by combining PC1 and PC2 from SSM/PCA in AD patients and NCs in the identification cohort. Cool color indicates regions with decreased loading, and warm color indicates regions with increased loading. The pattern was overlaid onto a standard MRI brain template to display voxels that were reliable at *P* < 0.001 based on the bootstrapping algorithm. MCC middle cingulate cortex, PCC posterior cingulate cortex

To identify the CBF pattern for AD, we applied SSM/PCA analysis to the MRI CBF maps from the AD and NC groups. Focusing on the major source of variance in the CBF maps, we initially restricted the multiple regression model to include the subject expression scores from the first 6 SSM principal components (PC). The model that included PC1and PC2 (variance accounted for 22.13% and 13.07%, and regression coefficient *β* =  − 0.822 and − 0.569, respectively) was the best for distinguishing patients with AD from NCs. An ADRP was produced by a linear combination of these two PCs, accounting for 25.64% of the total subject × voxel variance. Figure 1 shows the topography of the ADRP that was reliable at *P* < 0.001 based on the bootstrapping algorithm (1000 iterations) and characterized by relatively negative weights in the bilateral middle and posterior cingulate and precuneus, bilateral inferior parietal lobule, and bilateral middle frontal gyrus, along with relatively positive weights in bilateral putamen and a few frontal areas. The locations of all brain regions and coordinates for voxels with local minimal and maximal weights for this ADRP are shown in Table 2.

**Table 2:** Regions involved in topographies of ADRP

Structure	BA	*X*	*Y*	*Z*	*T* value	Cluster size (ml)
Negative loading						
Bilateral middle cingulate^a^	23, 31	2	− 47	33	3.4	260.9
Bilateral precuneus^a^	7, 39, 40	2	− 63	36	3.0	^b^
Right middle frontal gyrus	8, 9	39	23	38	1.5	13.7
Left middle frontal gyrus	8, 9	− 29	35	38	1.7	12.7
Left cerebellum		− 21	− 77	− 23	1.3	3.7
Positive loading						
Right superior frontal gyrus	11	3	60	− 28	2.1	149.9
Right frontal orbital gyrus	11	6	54	− 33	2.0	^b^
Left frontal rectal gyrus	11	− 3	55	− 31	2.0	^b^

The coordinates were reliable at *P* < 0.001 based on the bootstrapping algorithm. Cluster size > 100

*ADRP* AD-related perfusion pattern, *BA* Brodmann area

^a^This region extends to and involves the bilateral posteior cingulate and inferior parietal lobule according to the overlapped map

^b^Region belongs to the cluster for which the size is provided above

### Voxel-based CBF changes from univariate analysis

**Fig. 2:**
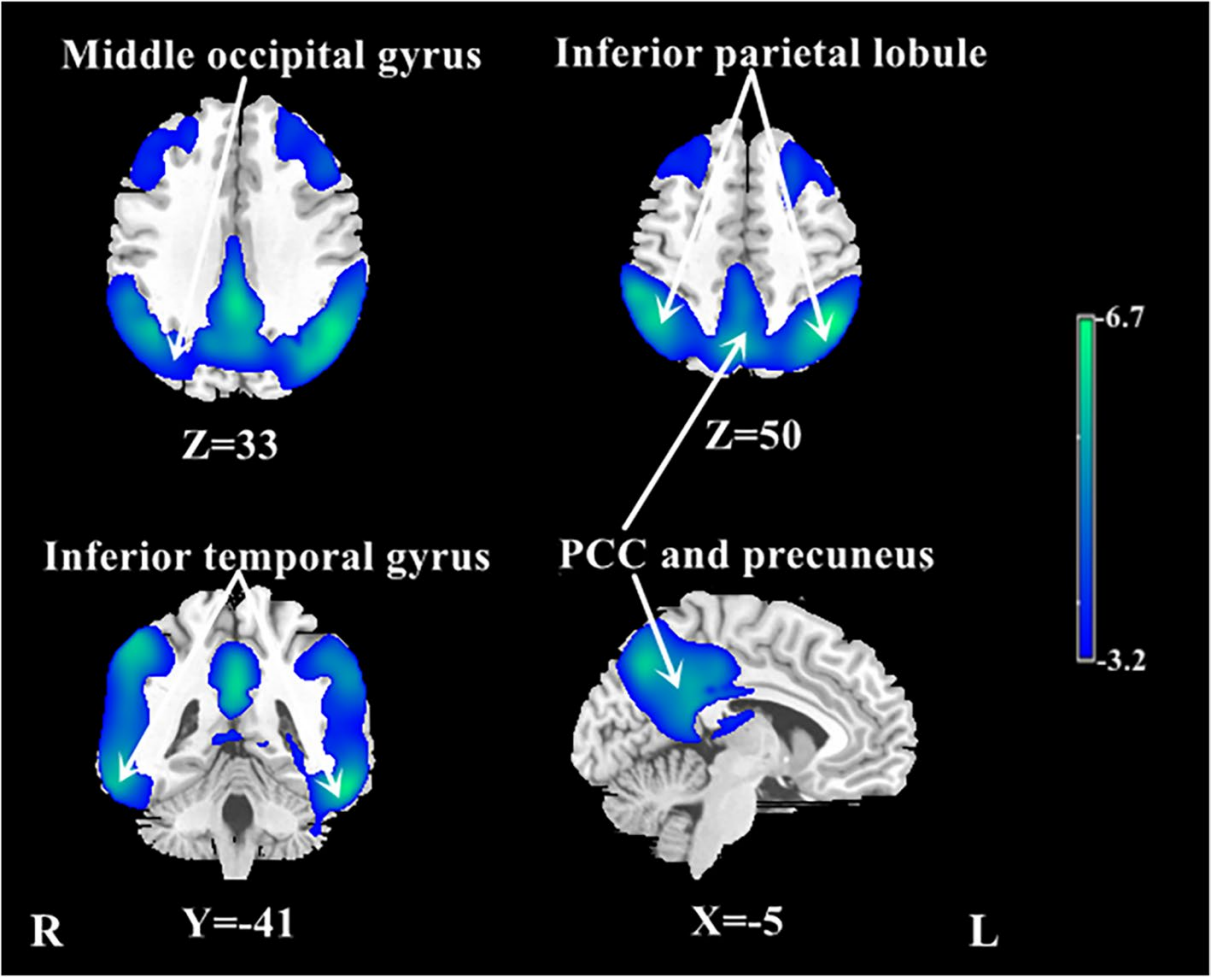
Regional CBF changes in patients with AD from univariate analysis without adjusting for the global value in the identification cohort. Cool color indicates regions with decreased CBF in AD patients compared with NCs. A threshold of 3.23 (*P* < 0.001, uncorrected) was used to overlay SPM maps onto a standard MRI brain template. PCC posterior cingulate cortex

We reported all significant clusters in SPM analysis with a voxel-level peak threshold of *P* < 0.05 (FWE-corrected) over whole brain regions. Without adjusting for the global value, AD patients showed decreased absolute CBF in many brain areas, including the bilateral posterior cingulate, bilateral precuneus, bilateral inferior parietal lobule, bilateral inferior temporal gyrus, right superior temporal gyrus, right middle temporal gyrus, and right middle occipital gyrus, relative to the NC group (Table 3, Fig. 2).

**Table 3:** Regions showing reduced absolute CBF in patients with AD (without normalization)

Structure	BA	*X*	*Y*	*Z*	*T*	Cluster size (ml)
Bilateral posterior cingulate	23, 31	− 2	− 45	33	6.3	29.3
Bilateral precuneus	7	2	− 68	44	6.1	^a^
Left inferior parietal lobule	40	− 47	− 54	47	6.7	51.8
Right inferior parietal lobule	39, 40	54	− 39	57	6.2	42.3
Left inferior temporal gyrus	20, 37	− 57	− 42	− 20	6.6	51.8
Right inferior temporal gyrus	20	57	− 44	− 12	5.9	42.3
Right middle temporal gyrus	21	66	− 27	− 12	5.8	^a^
Right superior temporal gyrus	42	36	− 57	33	5.4	^a^
Right middle occipital gyrus	19, 37	38	− 71	8	5.1	^a^

Significant clusters were defined using an FWE correction at *P* < 0.05

*BA* Brodmann area

^a^Regions belong to the same cluster for which the volume is provided above

**Fig. 3:**
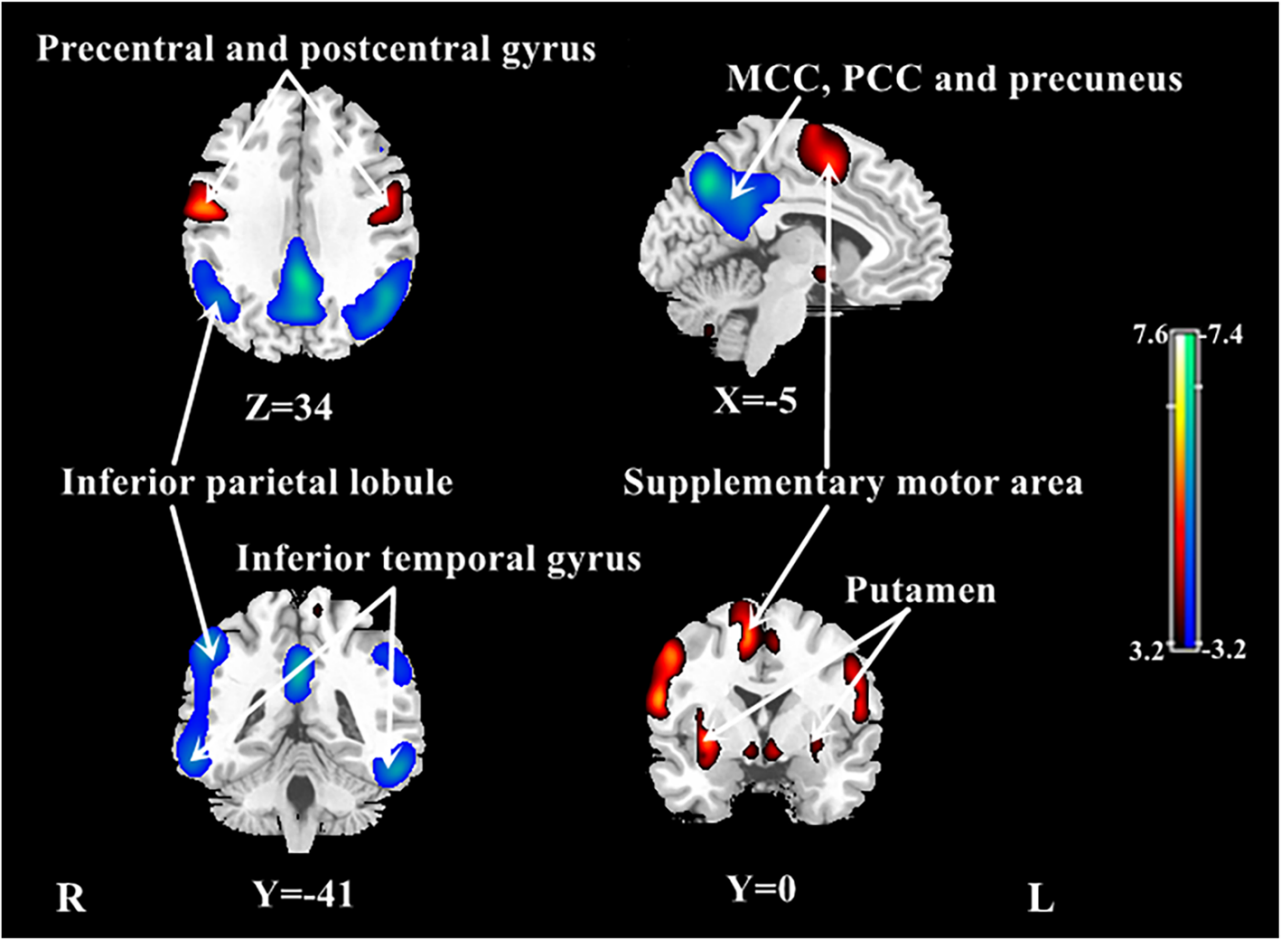
Regional changes in relative CBF after ANCOVA normalization for the global value in patients with AD in the identification cohort. Cool color indicates regions with relative decreased CBF, and warm color indicates regions with relative increased CBF in AD patients compared to NCs. A threshold of 3.23 (*P* < 0.001, uncorrected) was used to overlay SPM maps onto a standard MRI brain template. MCC middle cingulate cortex, PCC posterior cingulate cortex

After ANCOVA normalization for the global value, both relatively decreased and relatively increased CBF were observed in the AD group in different regions (Table 4, Fig. 3). CBF in the bilateral posterior cingulate, bilateral middle cingulate, bilateral precuneus, bilateral inferior parietal lobule, and bilateral inferior temporal gyrus was relatively reduced in patients with AD compared to NCs. Regions with relatively increased CBF in the AD group included the right parietal postcentral gyrus, bilateral frontal precentral gyrus, right frontal supplementary motor area, and right putamen.

**Table 4:** Regions showing relative CBF changes in patients with AD compared to NCs after adjusting for global value (ANCOVA normalization)

Structure	BA	*X*	*Y*	*Z*	*T*	Cluster size (ml)
Decreased CBF						
Bilateral posterior cingulate	23, 31	− 2	− 48	32	7.1	11.5
Bilateral middle cingulate	23	2	− 33	41	5.5	^a^
Bilateral precuneus	7	3	− 66	44	7.4	^a^
Right inferior parietal lobule	39, 40	53	− 41	51	5.9	4.0
Right inferior temporal gyrus	20	59	− 44	− 15	5.3	0.6
Left inferior parietal lobule	7, 40	− 45	− 51	47	7.0	9.1
Left inferior temporal gyrus	20, 37	− 56	− 42	− 20	6.0	1.3
Increased CBF						
Right parietal postcentral gyrus	43	59	− 6	23	7.3	7.8
Right frontal precentral gyrus	4, 6	38	− 18	57	7.5	^a^
Right frontal supplementary motor area	6	9	3	53	6.3	1.2
Left frontal precentral gyrus	4, 6	− 53	3	24	5.6	0.5
Right putamen		32	2	0	6.0	1.2

Significant clusters were defined using an FWE correction at *P* < 0.05

*BA* Brodmann area

^a^Region belongs to the cluster for which the volume is provided above

### Discrimination of AD patients from normal controls by ADRP subject expression and CBF

**Fig. 4:**
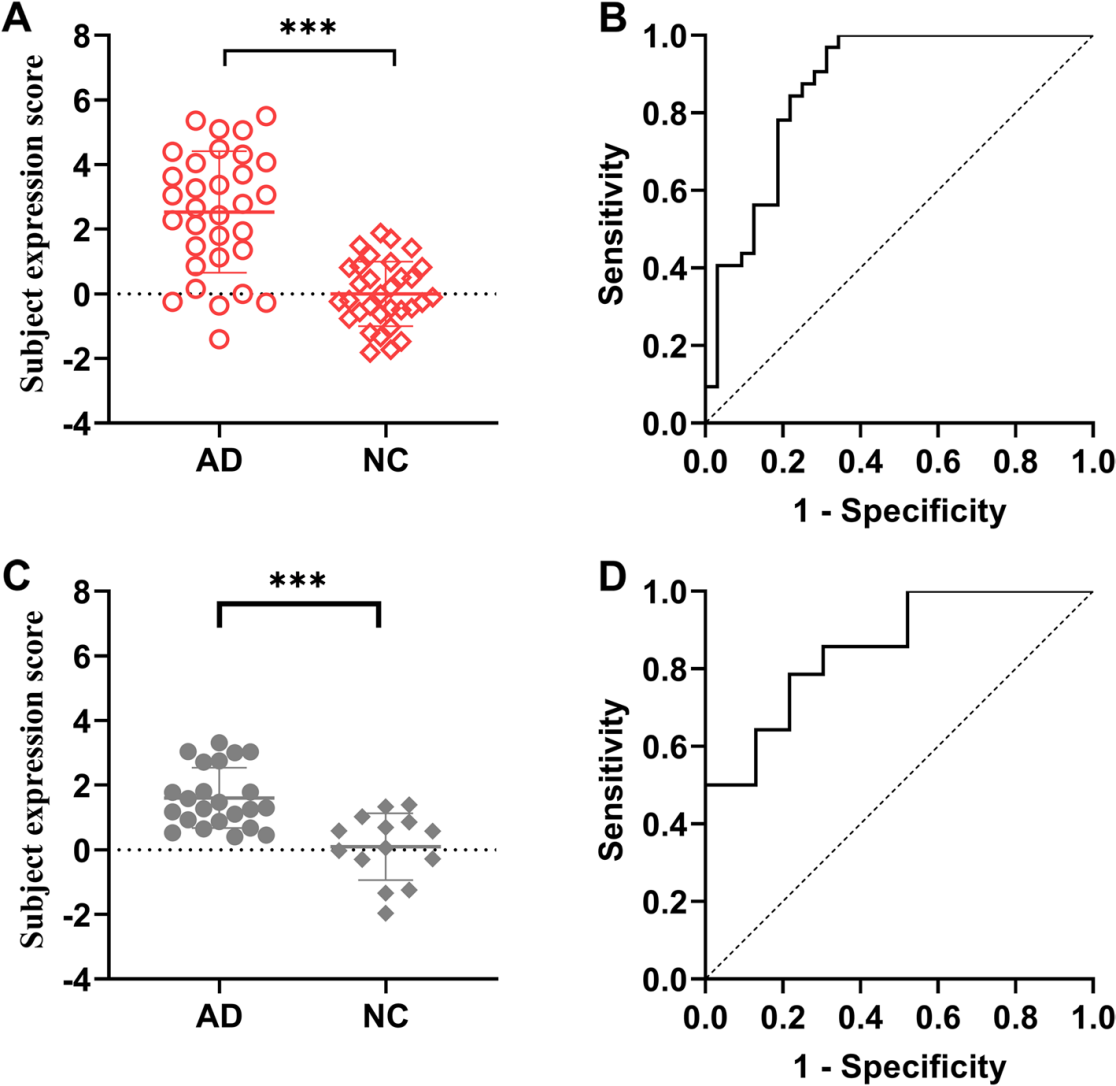
Subject expression score of ADRP in distinguishing patients with AD from NCs. **A** Comparison of subject expression score between the AD group and the NC group in the identification cohort. **B** ROC curve of subject expression score for discrimination between patients with AD and NCs. The AUC value was 0.87 at a cutoff of 1.7 in subject expression score, with a sensitivity of 68.75% and a specificity of 96.88%. In the validation cohort, comparison of subject expression score (**C**) and its ROC curve (**D**) for discrimination between patients with AD and NCs. The AUC value was 0.85 at a cutoff of 0.9 in subject expression score, with a sensitivity of 78.26% and a specificity of 78.57%. ***P < 0. 001. ADRP AD-related CBF pattern, ROC receiver operating characteristic, AUC area under the curve

Subject expression of the ADRP showed a significant elevation in AD patients compared to NCs in the identification cohort (*t* = 6.740, *P* < 0.001, Fig. 4A). An ROC curve was generated for subject expression scores of the ADRP to determine the optimal cutoff value in distinguishing patients with AD from NCs (Fig. 4B). The area under the ROC curve was 0.87 (95% confidence interval 0.78–0.96). At an optimal cutoff value of 1.7, subject expression score of the ADRP showed a sensitivity of 68.75%, a specificity of 96.88%, a positive predictive value of 0.96 (22 true-positive and 1 false-positive findings), and a negative predictive value of 0.76 (31 true-negative and 10 false-negative findings) for distinguishing AD patients from NCs. For the validation cohort, subject expression score of the ADRP was also significantly elevated in AD patients compared to NCs (*t* = 4.573, *P* < 0.001, Fig. 4C) and had an area under the ROC curve of 0.85 (95% confidence interval 0.73–0.98), showing a sensitivity of 78.26% and a specificity of 78.57% at an optimal cutoff value of 0.9 (Fig. 4D).

**Fig. 5:**
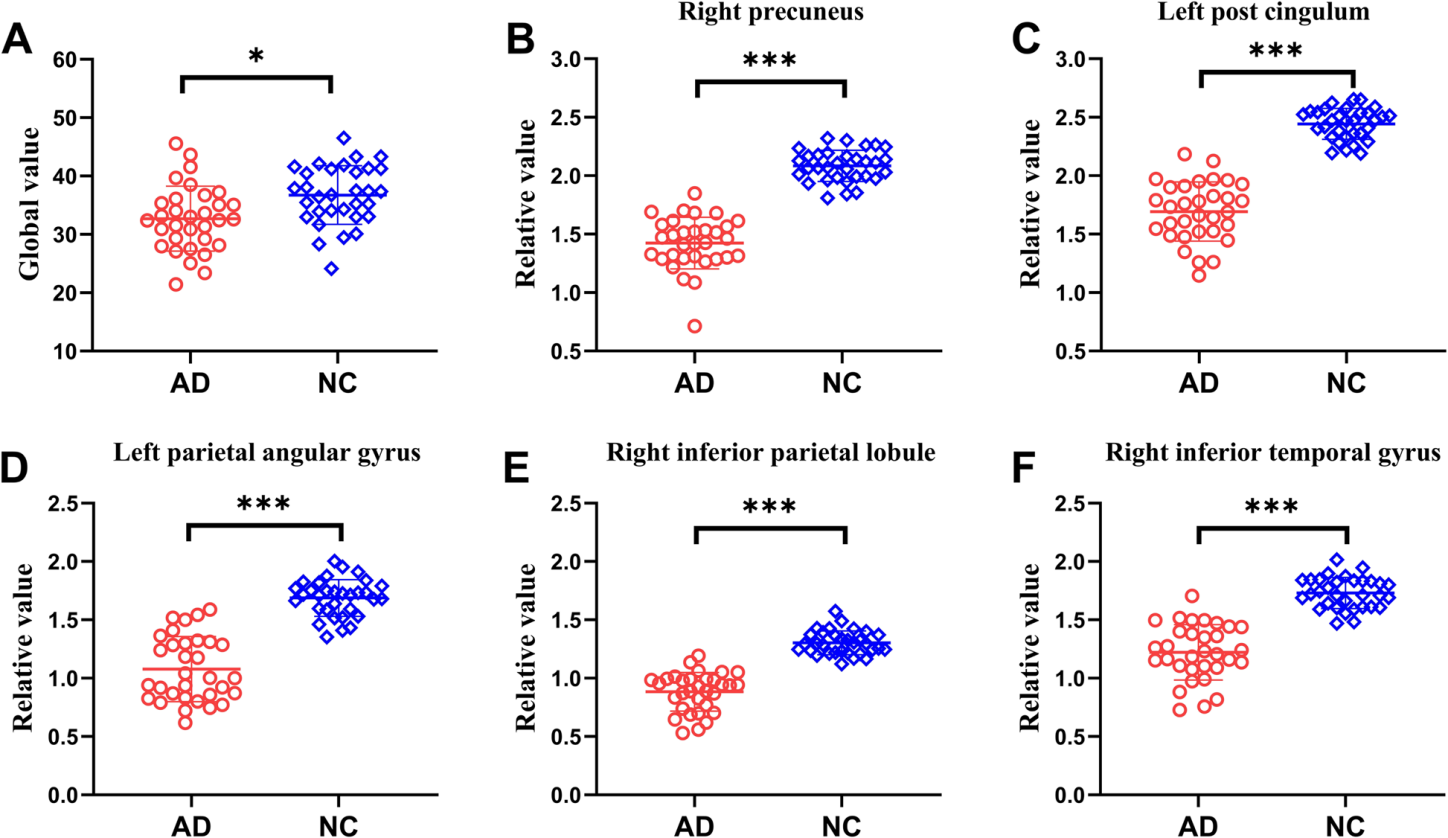
Difference in global CBF value and relative CBF values for five sample regions between the AD group and the NC group in the identification cohort. **A** The comparison of global value between AD patients and NCs from the CBF map. **B–F** The comparisons of relative values in the right precuneus (3, − 66, 44), left posterior cingulate (− 2, − 48, 32), left inferior parietal lobule (− 36, − 62, 45), right inferior parietal lobule (53, − 41, 51), and right inferior temporal gyrus (59, − 44, − 15) between AD patients and NCs, obtained post hoc within a spherical volume of interest (4 mm radius). *P < 0.01; ***P < 0.001

Global value measured from the CBF map was significantly reduced in the AD group compared to NCs (32.7 ± 5.6 vs 36.7 ± 5.0 ml/100 g/min, *t* = 3.065, *P* < 0.01; Fig. 5A). Sample plots for the major regions with decreased relative CBF in the AD group, including the right precuneus, left posterior cingulate, left inferior parietal lobule, right inferior parietal lobule, and right inferior temporal gyrus, are shown in Fig. 5B–F. ROC curves were generated for both global and relative regional CBF values in distinguishing patients with AD from NCs (Supplementary Fig. 1). The area under the ROC curve was 0.72 (95% confidence interval 0.60–0.85), with a sensitivity of 65.63%, a specificity of 71.88% for global CBF value. In terms of relative regional CBF values, the left posterior cingulate showed the highest sensitivity (100%) and specificity (100%) for differentiating patients with AD from NCs.

### Cognitive correlations of ADRP subject expression and CBF in AD patients

**Fig. 6:**
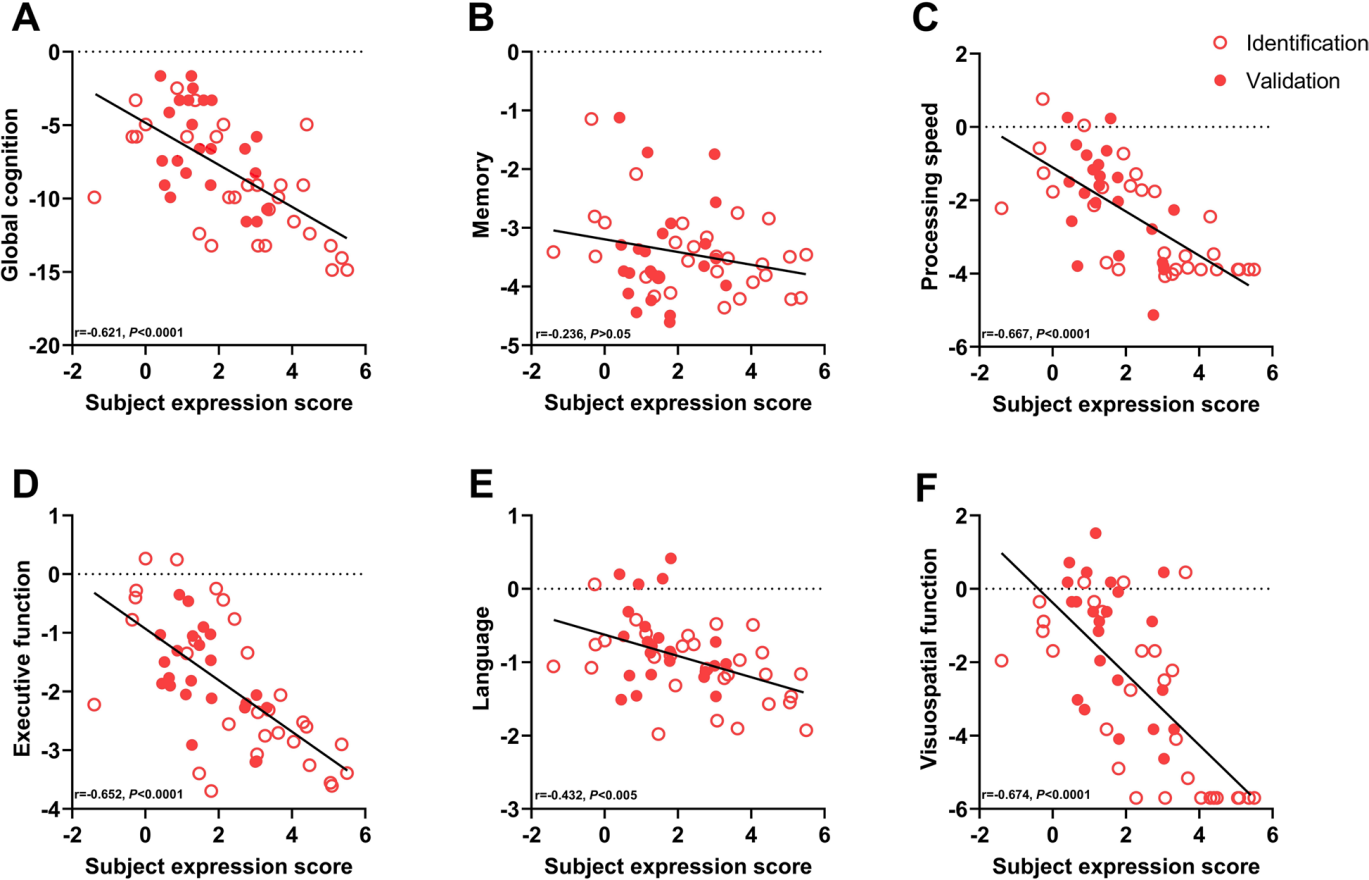
The correlation between ADRP subject expression and cognitive function in patients with AD. Subject expression score negatively correlated with global cognition measured with the MMSE (**A**), and various cognitive domains, including attention and information processing speed (**C**), executive function (**D**), language (**E**), and visuospatial function (**F**) in all AD patients from both the identification cohort and the validation cohort. There was no correlation between subject expression score and memory (**B**) domain in all AD patients, although a negative correlation was shown in the identification cohort. Raw scores were converted to z-scores for both global cognition and different cognitive domains. Data for cognitive domains was missing from 3 AD patients in the identification cohort

Subject expression scores of the ADRP negatively correlated strongly with the z-score of both global cognition and the composite z-scores of all cognitive domains in AD patients from the identification cohort and negatively correlated moderately with all cognitive domains except memory and language in the validation cohort (Table 5, Fig. 6). Relative CBF values in VOI regions, but not global CBF values, showed positive correlations with cognitive function to some extent in AD patients (Table 5).

**Table 5:** Correlation between subject expression scores of ADRP, global CBF and relative CBF of VOI, and cognitive function in patients with AD

	ADRP subject score				CBF											
	Identification cohort		Validation cohort		Global		Right precuneus		Left posterior cingulate		Left angular		Right inferior parietal lobule		Right inferior temporal gyrus	
	*r*	*P*	*r*	*P*	*r*	*P*	*r*	*P*	*r*	*P*	*r*	*P*	*r*	*P*	*r*	*P*
Global cognition	− 0.637	< 0.001	− 0.441	0.035	0.239	0.213	0.434	0.019	0.619	< 0.001	0.537	0.003	0.475	0.009	0.361	0.054
Memory	− 0.430	0.020	0.055	0.805	0.218	0.256	0.503	0.005	0.470	0.010	0.438	0.018	0.435	0.018	0.426	0.021
Processing speed	− 0.716	< 0.001	− 0.599	0.003	0.246	0.198	0.502	0.006	0.645	< 0.001	0.602	< 0.001	0.498	0.006	0.483	0.008
Executive function	− 0.674	< 0.001	− 0.559	0.006	0.237	0.215	0.436	0.018	0.577	0.001	0.522	0.004	0.563	0.002	0.530	0.003
Language	− 0.464	0.011	− 0.270	0.213	0.352	0.061	0.230	0.231	0.434	0.019	0.506	0.005	0.312	0.099	0.293	0.123
Visuospatial function	− 0.696	< 0.001	− 0.478	0.021	0.232	0.227	0.338	0.073	0.573	0.001	0.488	0.007	0.708	< 0.001	0.589	< 0.001

z-scores for global cognition and different cognitive domains were calculated from raw scores of neuropsychological assessments referenced the mean and standard deviation of all NCs. Global cognition was measured with the MMSE. Memory = average of total learning, delayed recall and recognition on the AVLT and the BVMT-R; processing speed = average of the SDMT and the TMT-A; executive function = average of the TMT-B and the Stroop color-word test; language = average of the AFT, the COWAT and the BNT; visuospatial function = the JLO. Data for cognitive domains was missing from 3 AD patients of identification cohort. Correlation analysis for global and regional CBF were conducted in AD patients from identification cohort

## Discussion

This study established an AD-related CBF covariance pattern measured with ASL MRI using SSM/PCA. Consistent with the results of voxel-wise univariate analysis with normalization for global CBF value, the ADRP indicates a relatively negative loading in the bilateral middle and posterior cingulate and precuneus, bilateral inferior parietal lobule, and bilateral middle frontal gyrus, and a relatively positive loading in the bilateral putamen and a few frontal areas in patients with AD. Both subject expression scores of the ADRP and relative regional CBF value could efficiently distinguish patients with AD from healthy individuals. Moreover, compared with relative regional CBF, the ADRP subject expression scores more strongly correlated with global cognition and various cognitive domains, including memory, attention and information processing speed, executive function, language, and visuospatial ability.

To the best of our knowledge, this is the first study to establish an ADRP with SSM/PCA using pCASL data in patients with amyloid biomarker supported AD. This perfusion ADRP accounted for 25.64% of the variance in the CBF imaging data and included both negative and positive loadings. However, a previous ASL MRI study with a small sample only reported a covariance perfusion pattern characterized by negative loadings mostly around the parahippocampal gyrus in the medial temporal and occipital lobes, as well as the thalamus in clinically diagnosed patients with AD; but no areas with positive loadings were found using SSM/PCA [12]. Our ADRP is consistent with the results of multivariate analysis from an early study using the gold standard perfusion measure of H_2_O^15^ PET, which observed decreased concomitant flow with SSM/PCA in the bilateral inferior parietal lobule and cingulate, left middle and inferior frontal, and precentral and supramarginal gyri, although they found increased concomitant flow in more extensive areas than we observed, including the bilateral insula, lingual gyri and cuneus, left fusiform and superior occipital gyri, and right parahippocampal gyrus and pulvinar [25].

The metabolic covariance pattern measured with FDG PET was also characterized by negative loading in the posterior temporo-parietal and prefrontal regions and relatively positive loading in the subcortical, cerebellum, and sensorimotor regions in patients with AD [11]. The regions with negative loading in our ASL established ADRP are highly consistent with those in the signature FDG pattern for patients with typical AD [26]. The consistency of findings from ASL MRI and FDG PET supports a coupling between perfusion and metabolism. Since FDG PET is considered as a biomarker of neurodegeneration or neuronal injury in the pathological process of AD, our results suggest that ASL could be an alternative to FDG PET in the diagnosis and evaluation of patients with AD, at least at the mild to moderate stage.

Moreover, our ability to discriminate between AD and NCs on the basis of the perfusion ADRP subject expression scores is consistent with previous metabolic and perfusion studies with PET. Metabolic pattern measured with FDG PET exhibited a sensitivity of 82.0% and a specificity of 94.0% for discriminating AD from NCs using subject expression score [27]. Another study [28] found that the expression of a previously established AD-related metabolic pattern was significantly different between stable subjects and AD converters at baseline in a mild cognitive impairment (MCI) cohort, with an area under the ROC curve of 0.80. Furthermore, the abovementioned H_2_O^15^ PET study [25] showed that the expression of the perfusion covariance pattern had good diagnostic accuracy (sensitivity of 76% and specificity of 81%) in discriminating AD from NCs and could predict the decline of memory and cognitive performance in subjects with minimal to mild cognitive impairment. A recent study used PCA to identify a metabolic AD conversion-related pattern on FDG PET and found that the resulting pattern expression score was superior to clinical variables in predicting conversion from MCI to AD over a follow-up period of 5 years [29]. Our perfusion ADRP showed a modest sensitivity of 68.75% and a very high specificity of 96.88% for the identification cohort and good diagnostic performance (both sensitivity and specificity above 78%) for the validation cohort in distinguishing AD patients from the NCs. Of note, the mean ADRP score and optimal cutoff value were lower in the validation cohort because those AD patients had lower severity than those in the identification cohort based on the MMSE scores and scores of language and visuospatial function (Table 1), given the negative correlations (Fig. 6) between cognitive function and ADRP score in AD patients across both cohorts. Large samples with comparable disease severity and cross-validation would be needed to establish the reproducibility of ADRP topographies and its expression for disease discrimination.

Consistent with previous studies [6], we demonstrated robust global hypoperfusion, and extensive regional CBF reduction and corresponding cognitive correlations using univariate analysis in patients with AD at the mild to moderate stage, in particular the middle and posterior cingulate, precuneus, and temporo-parietal association cortex; and relative CBF value in these regions, in particular the left posterior cingulate, showed high sensitivity and high specificity in distinguishing AD patients from NCs. It is well-established that vascular dysfunction, including changes in the blood–brain barrier integrity and CBF, is a prominent and early feature in AD pathophysiology. Cerebral hypoperfusion could give rise to reduction in Aβ clearance and subsequent accumulation of amyloid plaques and neurofibrillary tangles; in turn, the core AD pathology might exacerbate vascular injury and CBF decline [30]. Moreover, measures of brain demyelination, which have been recently demonstrated to be important biomarkers for the pathology of AD and MCI [31], are also associated with CBF reduction. Since myelin homeostasis based on oligodendrocyte metabolism is an energy-consuming process, it is particularly sensitive to hypoxia, hypoperfusion, or ischemia [32].

In addition, we also found relatively increased regional CBF in a few brain areas of AD patients after adjusting for global value, such as frontal supplementary motor area, primary motor and sensory cortex, and subcortical deep GM. These areas are usually preserved in brain structure and function at the early stage of the neurodegenerative process in AD according to previous studies [33]. Interestingly, increased CBF was observed in various brain regions in patients with AD across different studies with or without normalizing CBF by a reference value such as global perfusion, including the insular cortex, temporal cortex, frontal cortex, anterior cingulate, hippocampus, amygdala, and basal ganglia [34–36], which was usually presumed to be a compensatory mechanism, especially at the MCI or mild dementia stage.

We noted the topographic similarity between regional loadings provided by SSM/PCA and perfusion differences revealed by SPM (cf. Figures 1 and 3). This is expected given that subject expression score of the ADRP is simply a summed product between the ADRP topography and an individual CBF image such that the greater the similarity between the ADRP and the CBF map, the higher the subject expression score in each AD patient [24]. Although relative CBF value in various regions had a high accuracy in distinguishing AD patients from NCs, subject expression score of the ADRP showed stronger and more extensive correlations with clinical variables, such as cognitive performance, in AD patients in this study. Of note, relative CBF values in this study were measured in spherical volumes of interest (4 mm radius) centered at the peak voxel of each significant cluster where group differences were maximal. The group differences would be smaller in conventional analytical methods owing to signal dilution from lower-intensity voxels when CBF values were measured over the entire cluster detected by SPM or the neuroanatomic structure defined on MRI. Because computation of pattern expression score is performed automatically without clinical information, this approach is more objective than diagnostic categorization achieved by visual interpretation or predefined region of interest (ROI) analysis [24]. Network analysis generally recovers more disease-specific, widely distributed brain regions that may not have direct biological correlates, whereas regional analysis is closer to the diagnostic process based on visual reading of clinical images [37]. According to our present results, it might be beneficial to combine the imaging markers obtained with these two complementary approaches to further improve the diagnosis and evaluation for AD.

## Limitations

All data used in this study were obtained from our longitudinal MRI research, in which the diagnosis of AD is supported by the core pathological biomarker of amyloid PET, and comprehensive neuropsychological testing and multimodal MRI were conducted for all participants. However, this study still has several limitations. First, like the majority of previous ASL studies, we reported CBF changes without correcting for partial volume effects (PVEs) given previous studies suggesting that correction for PVEs may not be necessary to improve the radiologic differentiation between patients with AD and subjects with subjective complaints [38], or to monitor disease severity in AD patients [39]. To examine any effect of brain atrophy on perfusion ADRP or CBF, we repeated the same SSM/PCA and SPM analysis with structural data in the identification cohort. Structural covariance pattern (ADRP-GM) was identified from a linear combination of the first 3 PC accounting for 14% of the total voxel × subject variance (Supplementary Fig. 2). We found that ADRP perfusion had small overlaps with a few atrophic regions in the ADRP-GM but only at the lowest level of reliability (*P* = 0.05) (Supplementary Fig. 3). Although there was some overlap between *t*-maps from CBF and GM tissue images without adjusting global value (Supplementary Fig. 4), it became minimal after adjusting for global difference in perfusion and GM tissue volume by ANCOVA even at the most liberal threshold of *P* = 0.05 (Supplementary Fig. 5). Hence, AD-related perfusion patterns in our study are truly vascular/perfusion-driven rather than a proxy measure for atrophy. Second, both SSM/PCA and SPM analyses were conducted within the same brain mask created from the high-resolution probabilistic GM map in SPM with a liberal threshold of pGM ≥ 0.3. To rule out any potential issues, we repeated SPM analysis in the identification cohort using the brain mask at a moderate threshold (pGM ≥ 0.5). The results showed the same regions of relative hypo- and hyper-perfusion as described above (see the comparison in Supplementary Fig. 6), indicating that the use of pGM ≥ 0.3 was adequate in this study. Third, repeated analyses in increasingly larger subsamples should be performed in a future study of SSM/PCA in conjunction with SPM analysis to determine the smallest sample sizes necessary for robust spatial covariance analysis. Fourth, although participants who had obvious large vessel vascular disease measured by carotid duplex ultrasound, TCD, or cranial MR angiography were excluded, the CBF maps might not reflect the effects of pure functional/microvascular physiology in the brain when blood flowing towards the brain is slowed (e.g., prolonged arterial transit time (ATT)). Nonetheless, the regional differences in CBF persisted in our AD sample after global normalization, suggesting that these findings were not entirely vascular. Recognizing the heterogeneous distributions of ATT across the brain, advanced protocols that include multiple post-labeling delays to account for effects of spatial variation in ATT [40] could be considered in future investigations. Finally, other factors that might influence cerebral perfusion, such as apolipoprotein E (APOE) genotype and WMH, were not taken into account in our data analysis, although we excluded obvious cerebral large and small vessel disease by multimodal MRI and duplex ultrasound.

While there was a significant correlation between subject expression score of the pattern and performance in global cognition and various cognitive domains, we could not prove whether this ADRP predicts disease progression because of cross-sectional analysis in the current study. In addition, unique brain networks associated with different cognitive disabilities could be respectively identified by multivariate analysis, with stronger correlations than those between this ADRP and all cognitive domains in the present study. For instance, several metabolic PCs measured with FDG PET were previously established in AD patients using SSM/PCA [10], namely, PC1 (posterior cortices) correlated with naming and visuospatial abilities, PC2 (limbic structures from the Papez circuit, e.g., medial temporal regions, posterior and anterior cingulate cortex, thalamus) correlated with episodic memory, and PC3 (frontal, parietal, temporal, and posterior medial association cortices) correlated with executive and global cognitive functions.

## Conclusion

An AD-related perfusion covariance pattern featuring negative loading mainly in the bilateral temporo-parietal cortex, cingulate, and precuneus and relatively positive loading marginally in the subcortical deep GM and frontal areas has been identified by multivariate analysis based on ASL data, and its expression is characterized in relation to cognitive impairment in various domains. The diagnostic utility of this ADRP was replicated in an independent validation cohort. This ADRP may provide a promising MRI biomarker for AD diagnosis and monitoring on a prospective single-subject basis.

## Supplementary Information

**Additional file 1:** **Supplementary Fig. 1.** ROC curves of both global and relative regional CBF values for discrimination between patients with AD and NCs. The AUC value was 0.72 with a sensitivity of 65.63% and a specificity of 71.88% for global CBF, and was 0.998 (sensitivity 100.00% and specificity 96.88%), 1.00 (sensitivity 100.00% and a specificity 100.00%), 0.975 (sensitivity 96.88% and specificity 87.50%), 0.996 (sensitivity 96.88% and specificity 96.88%), and 0.979 (sensitivity 93.75% and specificity 96.88%) for right precuneus, left PCC, left angular, right inferior parietal lobule, and right inferior temporal gyrus, respectively. ROC: receiver operator characteristic; CBF: cerebral blood flow; AUC: area under the curve; PCC: posterior cingulate cortex. **Supplementary Fig. 2.** AD-related covariance patterns (grey matter vs perfusion) in the identification cohort at different levels of reliability at each voxel. Structural covariance pattern (ADRP-GM) was identified from a linear combination of the first 3 principal components (PCs: variance accounting for = 11.5%, 6.9% and 3.6% respectively) accounting for 14% of the total voxel × subject variance. Brain regions in the ADRP-perfusion (displayed at a threshold value with high reliability of *P* = 0.001 as reported in the manuscript) was compared with their counterparts in the ADRP-GM displayed at threshold values corresponding to low, moderate and high levels of reliability (*P* = 0.05, 0.01 and 0.001) following the bootstrap test with 1000 iterations. **Supplementary Fig. 3.** The overlap of regional topographies with decreased loading of ADRP-CBF and ADRP-GM identified from SSM/PCA in the identification cohort. Blue color indicates regions with decreased loading in CBF, orange color indicates regions with decreased loading in gray matter volume, and pink color indicates regions with overlap of decreased loading in CBF and gray matter volume. The structural changes were only observed in a few isolated areas of smaller anatomic extent compared with CBF changes, even at threshold values with the lowest reliability of *P* = 0.05. **Supplementary Fig. 4.** The overlap of regional changes in decreased CBF and gray matter atrophy without normalization for the differences in global value across all AD and healthy subjects in the identification cohort. Blue color indicates regions with decreased CBF, orange color indicates regions with gray matter atrophy and pink color indicates regions with overlap of decreased CBF and gray matter atrophy in AD patients compared to NCs. To rule out false positives that were more pronounced in the results without global normalization, a stringent threshold of 4.67 or 5.56 (both at *P* < 0.05, FWE-corrected) for decreased CBF or gray matter atrophy was used to overlay SPM maps onto a standard MRI brain template. **Supplementary Fig. 5.** The overlap of regional changes in relative decreased CBF and gray matter atrophy after ANCOVA normalization for the differences in global value across all AD and healthy subjects in the identification cohort. Blue color indicates regions with decreased CBF, orange color indicates regions with gray matter atrophy, and pink color indicates regions with overlap of decreased CBF and gray matter atrophy in AD patients compared to NCs. To better appreciate relevant brain regions involved in the results, a liberal threshold of 1.67 (*P* < 0.05, uncorrected) was used to overlay both SPM maps onto a standard MRI brain template. **Supplementary Fig. 6.** Brain regions of abnormal perfusion (AD vs NC) in the identification cohort using masks with different threshold. SPM analysis was repeated in the identification cohort using the brain mask at a compromise threshold (pGM ≥ 0.5). The same regions of relative hypo- and hyper-perfusion were identified (despite slightly smaller extent) as those found with the brain mask of pGM ≥ 0.3 used in the article. A threshold of 3.23 (*P* < 0.001, uncorrected) was used to overlay SPM maps onto a standard MRI brain template.

## Data Availability

The datasets generated and analyzed during the current study are available from the corresponding author on reasonable request.

## Abbreviations

Aβ: Amyloid-β
AD: Alzheimer’s disease
ADRP: AD-related perfusion pattern
AFT: Animal Verbal Fluency Test
APOE: Apolipoprotein E
ASL: Arterial spin labeling
ATT: Arterial transit time
AUC: Area under the curve
AVLT: Auditory Verbal Learning Test
BNT: Boston Naming Test
bvFTD: Behavioral variant frontotemporal dementia
BVMT-R: Brief Visuospatial Memory Test-Revised
CBF: Cerebral blood flow
CDR: Clinical Dementia Rating
COWAT: Controlled Oral Word Association Test
CSF: Cerebrospinal fluid
DLB: Dementia with Lewy bodies
FA: Flip angle
FDG: Fluorodeoxyglucose
FOV: Field of view
FWHM: Full width at half maximum
GM: Grey matter
HAM-D: Hamilton Depression Scale
JLO: Benton Judgment of Line Orientation
MCI: Mild cognitive impairment
MMSE: Mini-Mental State Examination
MNI: Montreal Neurological Institute
NC: Normal control
NIA-AA: National Institute on Aging and the Alzheimer’s Association
pCASL: Pseudo-continuous ASL
PC: Principal components
PCA: Principal component analysis
PET: Positron emission tomography
PiB: Pittsburgh Compound B
PLD: Post labeling delay
PVEs: Partial volume effects
RBD: Rapid eye movement sleep behavior disorder
ROC: Receiver operator characteristic
ROI: Region of interest
SDMT: Symbol Digit Modalities Test
SPECT: Single photon emission computed tomography
SPM: Statistical parametric mapping
SSM: Scaled subprofile model
TCD: Transcranial doppler
TE/TR: Echo time/repetition time
TMT: Trail Making Test
VOI: Volume of interest
WM: White matter
WMH: White matter hyperintensities
