# Supplementary figures and images for: The identification and cognitive correlation of perfusion patterns measured with arterial spin labeling MRI in Alzheimer’s disease
Source: Alzheimers Res Ther. 2023 Apr 10;15:75. doi: 10.1186/s13195-023-01222-9 (PMC10088108; doi:10.1186/s13195-023-01222-9)

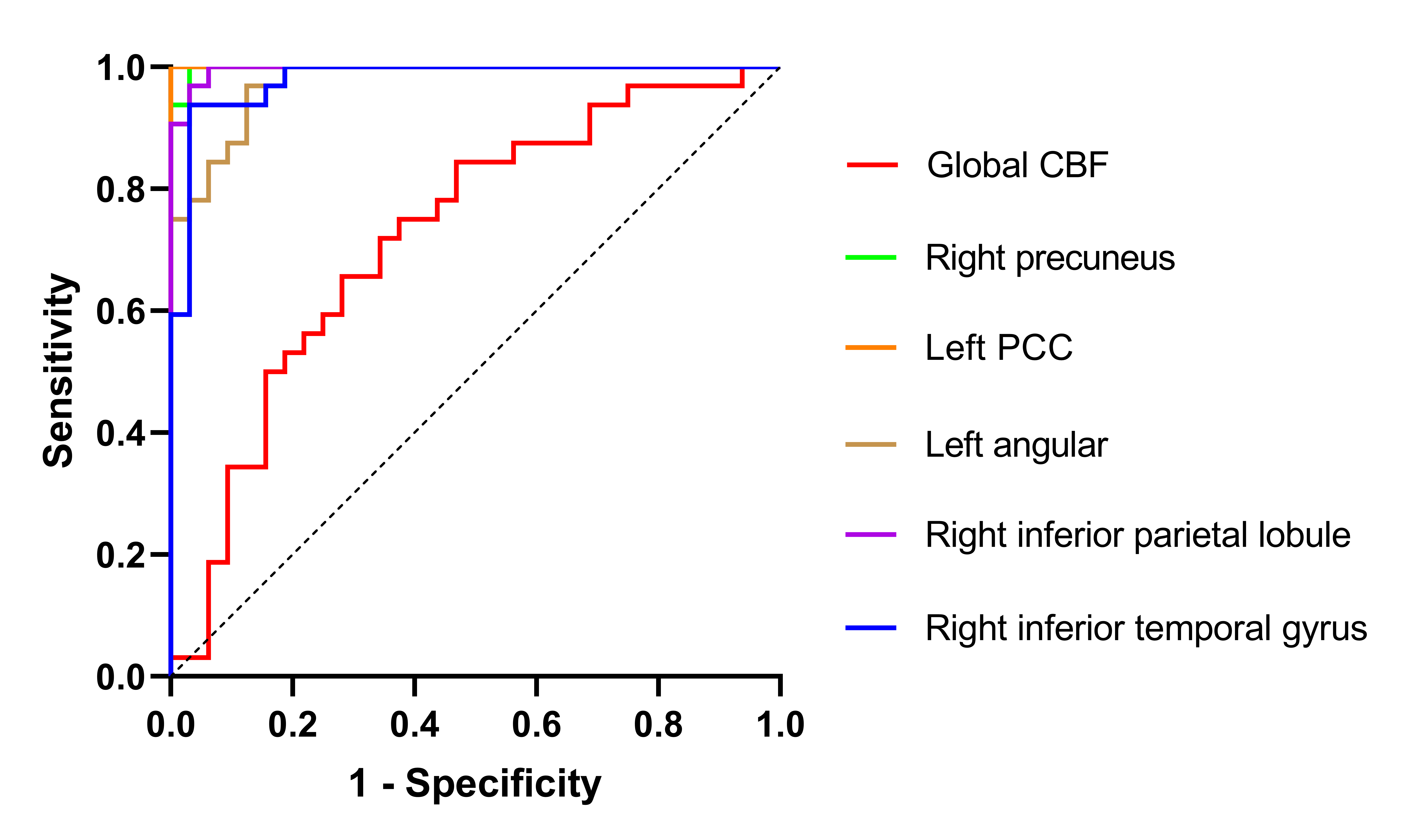

Supplement: Supplementary file 1 — Additional file 1: Supplementary Fig. 1. ROC curves of both global and relative regional CBF values for discrimination between patients with AD and NCs. The AUC value was 0.72 with a sensitivity of 65.63% and a specificity of 71.88% for global CBF, and was 0.998 (sensitivity 100.00% and specificity 96.88%), 1.00 (sensitivity 100.00% and a specificity 100.00%), 0.975 (sensitivity 96.88% and specificity 87.50%), 0.996 (sensitivity 96.88% and specificity 96.88%), and 0.979 (sensitivity 93.75% and specificity 96.88%) for right precuneus, left PCC, left angular, right inferior parietal lobule, and right inferior temporal gyrus, respectively. ROC: receiver operator characteristic; CBF: cerebral blood flow; AUC: area under the curve; PCC: posterior cingulate cortex. Supplementary Fig. 2. AD-related covariance patterns (grey matter vs perfusion) in the identification cohort at different levels of reliability at each voxel. Structural covariance pattern (ADRP-GM) was identified from a linear combination of the first 3 principal components (PCs: variance accounting for = 11.5%, 6.9% and 3.6% respectively) accounting for 14% of the total voxel × subject variance. Brain regions in the ADRP-perfusion (displayed at a threshold value with high reliability of P = 0.001 as reported in the manuscript) was compared with their counterparts in the ADRP-GM displayed at threshold values corresponding to low, moderate and high levels of reliability (P = 0.05, 0.01 and 0.001) following the bootstrap test with 1000 iterations. Supplementary Fig. 3. The overlap of regional topographies with decreased loading of ADRP-CBF and ADRP-GM identified from SSM/PCA in the identification cohort. Blue color indicates regions with decreased loading in CBF, orange color indicates regions with decreased loading in gray matter volume, and pink color indicates regions with overlap of decreased loading in CBF and gray matter volume. The structural changes were only observed in a few isolated areas of smaller a [file 13195_2023_1222_MOESM1_ESM.zip › Supplementary Fig.1.tif]

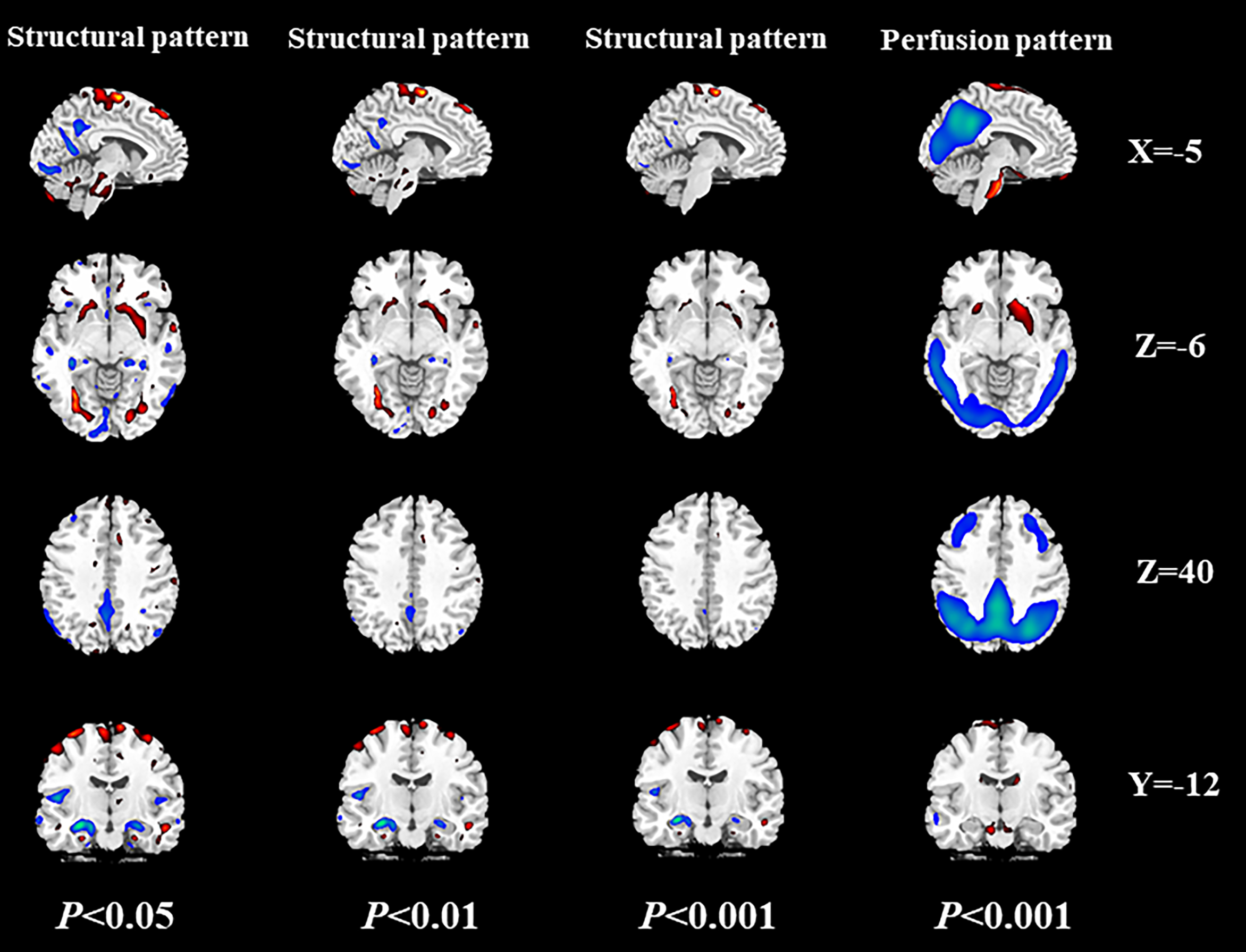

Supplement: Supplementary file 1 — Additional file 1: Supplementary Fig. 1. ROC curves of both global and relative regional CBF values for discrimination between patients with AD and NCs. The AUC value was 0.72 with a sensitivity of 65.63% and a specificity of 71.88% for global CBF, and was 0.998 (sensitivity 100.00% and specificity 96.88%), 1.00 (sensitivity 100.00% and a specificity 100.00%), 0.975 (sensitivity 96.88% and specificity 87.50%), 0.996 (sensitivity 96.88% and specificity 96.88%), and 0.979 (sensitivity 93.75% and specificity 96.88%) for right precuneus, left PCC, left angular, right inferior parietal lobule, and right inferior temporal gyrus, respectively. ROC: receiver operator characteristic; CBF: cerebral blood flow; AUC: area under the curve; PCC: posterior cingulate cortex. Supplementary Fig. 2. AD-related covariance patterns (grey matter vs perfusion) in the identification cohort at different levels of reliability at each voxel. Structural covariance pattern (ADRP-GM) was identified from a linear combination of the first 3 principal components (PCs: variance accounting for = 11.5%, 6.9% and 3.6% respectively) accounting for 14% of the total voxel × subject variance. Brain regions in the ADRP-perfusion (displayed at a threshold value with high reliability of P = 0.001 as reported in the manuscript) was compared with their counterparts in the ADRP-GM displayed at threshold values corresponding to low, moderate and high levels of reliability (P = 0.05, 0.01 and 0.001) following the bootstrap test with 1000 iterations. Supplementary Fig. 3. The overlap of regional topographies with decreased loading of ADRP-CBF and ADRP-GM identified from SSM/PCA in the identification cohort. Blue color indicates regions with decreased loading in CBF, orange color indicates regions with decreased loading in gray matter volume, and pink color indicates regions with overlap of decreased loading in CBF and gray matter volume. The structural changes were only observed in a few isolated areas of smaller a [file 13195_2023_1222_MOESM1_ESM.zip › Supplementary Fig.2.tif]

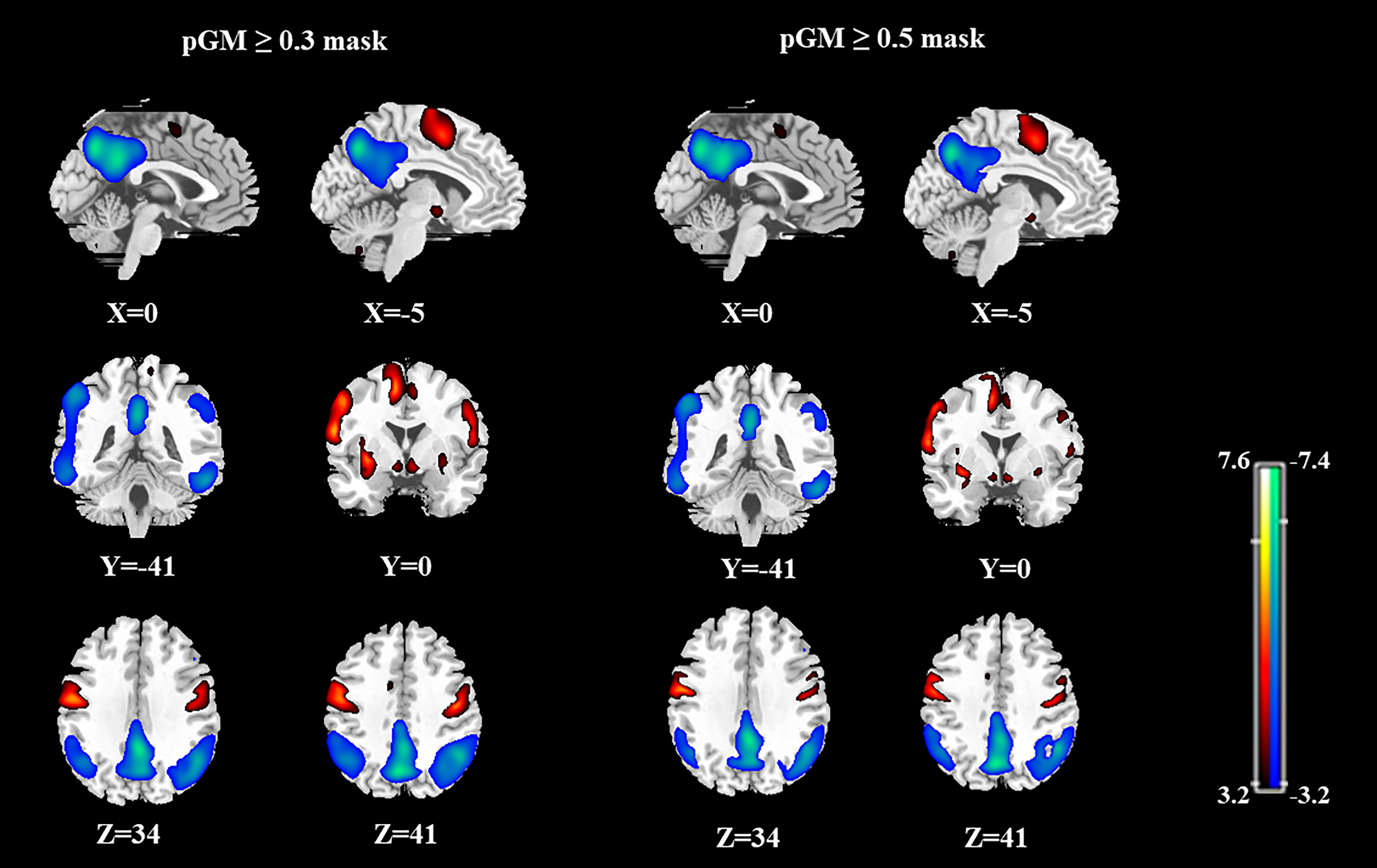

Supplement: Supplementary file 1 — Additional file 1: Supplementary Fig. 1. ROC curves of both global and relative regional CBF values for discrimination between patients with AD and NCs. The AUC value was 0.72 with a sensitivity of 65.63% and a specificity of 71.88% for global CBF, and was 0.998 (sensitivity 100.00% and specificity 96.88%), 1.00 (sensitivity 100.00% and a specificity 100.00%), 0.975 (sensitivity 96.88% and specificity 87.50%), 0.996 (sensitivity 96.88% and specificity 96.88%), and 0.979 (sensitivity 93.75% and specificity 96.88%) for right precuneus, left PCC, left angular, right inferior parietal lobule, and right inferior temporal gyrus, respectively. ROC: receiver operator characteristic; CBF: cerebral blood flow; AUC: area under the curve; PCC: posterior cingulate cortex. Supplementary Fig. 2. AD-related covariance patterns (grey matter vs perfusion) in the identification cohort at different levels of reliability at each voxel. Structural covariance pattern (ADRP-GM) was identified from a linear combination of the first 3 principal components (PCs: variance accounting for = 11.5%, 6.9% and 3.6% respectively) accounting for 14% of the total voxel × subject variance. Brain regions in the ADRP-perfusion (displayed at a threshold value with high reliability of P = 0.001 as reported in the manuscript) was compared with their counterparts in the ADRP-GM displayed at threshold values corresponding to low, moderate and high levels of reliability (P = 0.05, 0.01 and 0.001) following the bootstrap test with 1000 iterations. Supplementary Fig. 3. The overlap of regional topographies with decreased loading of ADRP-CBF and ADRP-GM identified from SSM/PCA in the identification cohort. Blue color indicates regions with decreased loading in CBF, orange color indicates regions with decreased loading in gray matter volume, and pink color indicates regions with overlap of decreased loading in CBF and gray matter volume. The structural changes were only observed in a few isolated areas of smaller a [file 13195_2023_1222_MOESM1_ESM.zip › Supplementary Fig.6.tif]
